# The Human IL-23 Decoy Receptor Inhibits T-Cells Producing IL-17 by Genetically Engineered Mesenchymal Stem Cells

**DOI:** 10.1155/2018/8213912

**Published:** 2018-12-19

**Authors:** Masoumeh Rostami, Kamran Haidari, Majid Shahbazi

**Affiliations:** ^1^Medical Cellular and Molecular Research Center, Golestan University of Medical Sciences, Gorgan, Iran; ^2^Department of Anatomy, Faculty of Medical Sciences, Golestan University of Medical Sciences, Gorgan, Iran

## Abstract

The immunomodulatory and self-renewable features of human adipose mesenchymal stem cells (hAD-MSCs) mark their importance in regenerative medicine. Interleukin 23 (IL- 23) as a proinflammatory cytokine suppresses T regulatory cells (Treg) and promotes the response of T helper 17 (Th17) and T helper 1 (Th1) cells. This pathway starts inflammation and immunosuppression in several autoimmune diseases. The current study for producing recombinant IL- 23 decoy receptor (RIL- 23R) using hAD-MSCs as a good candidate for ex vivo cell-based gene therapy purposes reducing inflammation in autoimmune diseases. hAD-MSCs was isolated from lipoaspirate and then characterized by differentiation. RIL- 23R was designed and cloned into a pCDH-813A- 1 lentiviral vector. The transduction of hAD-MSCs was performed at MOI (multiplicity of infection) = 50 with pCDH- EFI *α*- RIL- 23R- PGK copGFP. Expressions of RIL- 23R and octamer-binding transcription factor 4 (OCT- 4) were determined by real-time polymerase chain reaction (real time-PCR). Self-renewing properties were assayed with OCT- 4. Bioactivity of the designed RIL- 23R was evaluated by IL- 17 and IL- 10 expression of mouse splenocytes. Cell differentiation confirmed the true isolation of hAD-MSCs from lipoaspirate. Restriction of the enzyme digestion and sequencing verified the successful cloning of RIL- 23R in the CD813A-1 lentiviral vector. The green fluorescent protein (GFP) positive transduction rate was up to 90%, and real-time PCR showed the expression level of RIL-23R. Oct-4 had a similar expression pattern with nontransduced hAD-MSCs and transduced hAD-MSCs/ RIL-23R indicating that lentiviral vector did not affect hAD-MSCs characteristics. Downregulation of IL-17 and upregulation of IL-10 showed the correct activity of the engineered hAD-MSCs. The results showed that the transduced hAD-MSCs/ RIL- 23R, expressing IL-23 decoy receptor, can give a useful approach for a basic research on cell-based gene therapy for autoimmune disorders.

## 1. Introduction

The mesenchymal stem cells (MSCs) are one of the adult stem cells capable of being differentiating into mesoderm-lineage cells, including osteoblasts, chondrocytes, and adipocytes. They are characterized by attachment to plastic culture vessels and the ability to express the CD44, CD73, CD90, and CD105 but not CD45, CD34, and CD14 cell surface markers [1]. Many studies have found that MSCs have both hypoimmunogenic and immunomodulatory properties that allow them to home to damaged tissues and initiate healing through repair processes [2–4]. These characteristics of MSCs support the idea that the creation of genetically modified MSCs could be the best option for combining cell and gene therapy to treat diverse forms of autoimmune diseases [5–10].

Among which, the Adipose-MSCs (AD-MSCs) are a rich source for MSCs in the therapeutic purposes as adipose tissue is easily accessible and large amounts of AD-MSCs are easily obtainable [11–13]. Currently, AD-MSCs are clinically applied for regenerative treatments and wound healing [14, 15]. Thus, the ability of human adipose-derived MSCs to serve as vehicles for a cell-based gene therapy is promising [16–19].

Autoimmune diseases are multi-factorial disorders with complicated immune system dysregulation mediated by immune cytokines and immune cells [20]. The IL- 23 belonging to the family IL-12 plays an active role to proliferate the memory T helper 1 cells. The hetero dimerized IL- 23 receptor constituted of specific (IL23A) and common (IL12R*β*1) subunits [21]. The transforming growth factor beta (TGF-*β*) and IL-6 in most of the autoimmune diseases can induce Th17 cells to secrete increasingly IL-23 and IL-17 [22]. The IL-23 is able to suppress the Treg cells and promote the response of Th17 and Th1 cells, initiating inflammation and immunosuppression in several autoimmune and inflammatory diseases [23]. Based on the strong evidence, the IL- 23 /IL- 17 axis is important for the development of chronic inflammation [24]. Recent studies determined that the suppression of IL-23, the IL-23R, or the IL-23 /IL-17 axes potentially can be therapeutic targets for the autoimmune diseases [25]. However, there are no specific treatments for inhibition of the IL-23 proinflammatory responses.

Targeting IL-23p19, but not IL-12p40, in gene knockout studies showed that the decrease of proinflammatory responses and resistance to different autoimmune diseases are due to the absence of IL-17-producing T-cells (i.e., Th17 cells) [26].

Based on earlier studies, extensive alternative splicing exerted on the IL- 23R gene transcript [27]. Generation of IL- 23RΔ9 form (GenBank AM990318), which encodes a soluble version of the entire external domain of the specific receptor chain (IL23A), is an example of this splicing [28]. After binding human IL- 23 in solution, this soluble decoy receptor dependently inhibits STAT3 phosphorylation and functional maturation of human Th17 cells in vitro [29].

Based on the immunosuppressive functions of hAD-MSCs and RIL-23R, they can cooperate to improve the immunomodulatory and prevent the initiation of inflammation and latter autoimmune diseases. The current report was the first case of RIL-23R gene transduction into hAD-MSCs. This study successfully transduced hAD-MSCs by recombinant Interleukin 23R-harbouring lentiviral particles and evaluated the expression of RIL-23R in transduced hAD-MSCs by real time-PCR. It also analyzed the RIL-23R bioactivity and the effect of this cytokine on the T-cells.

## 2. Materials and Methods

### 2.1. Isolation of Mesenchymal Stem Cell from Human Adipose Tissue

Lipoaspirate samples were washed with phosphate-buffered saline (PBS) containing 3 X penicillin/ streptomycin and amphotericin three times. The adipose tissue was added dispase (50 u/ml)/ collagenase I (250 u/ml) (Sigma-Aldrich, St. Louis, MO), followed by shaking for 30 min at 37°C and then centrifugation at 1500 rpm. The plated cells after suspending were distributed in the flasks with *α*-MEM (minimum essential medium eagle-alpha modification) containing 10% fetal bovine serum (FBS) for three days.

### 2.2. Cloning of RIL- 23R cDNA into a Lentiviral Vector

IL-23 soluble form, IL-23RΔ9 (GenBank AM990318), which encodes a version of the entire external domain of the receptor chain, (alternative splicing product) was designed and amplified in the pORF plasmid (Biomatik, Cambridge, Ontario, Canada) using the Forward primer of 5′ ACAACAGCTCGGCTTTGGTAT -3′, and Reverse primer of 5′ TACTGGCAGCCTTGGAGTTC -3′.

RIL-23R-pORF-For synthesizing the digestion of GFP-harboring lentiviral particles (LvGFP), GFP-carrying vector was digested by EcorV and Sal1, cDNA subcloned into pCDH813A-1 (System Bioscience, Mountain View, CA, United States) to make a recombinant vector (pCDH-EF-1*α*–RIL- 23R-PGK-copGFP).

### 2.3. Generation, Concentration, and Titration of Recombinant Lentivirus

The recombinant lentivirus was produced according to the previous protocol [30, 31]. In summary, the transfection of plasmid DNA into 1 × 10^6^ HEK-293T cells was based on calcium–phosphate method (21 *μ*g of lentivirus involving pCDH- RIL-23R-copGFP, 21 *μ*g of packaging plasmid psPAX2, and 10.5 *μ*g of envelope plasmid per 10-cm plate). The lentivirus particles contained in the supernatant of HEK- 293 T cells were collected at 36, 48, 60, and 72 hours posttransfection and then passed through a 0.45 *μ*m filter. The concentration process of lentivirus particles was performed according to the precipitation method using 50% PEG-8000 (Sigma-Aldrich, St. Louis, MO, USA) to reach a final concentration of 5%. The concentration was continued with NaCl 5 M (Sigma-Aldrich, St. Louis, MO, USA) overnight to achieve a final concentration of 0.15 M. Then, 6 × 10^4^ HEK- 293T cells were plated into plates for titration, followed by transduction with 1, 4 and 16 *μ*l of the virus. After three days of incubation, the cells were detached and flow cytometry was used to analyze cell fluorescence.

### 2.4. Transduction of hAD-MSCs

The second passage hAD-MSCs were trypsinized and were seeded in 6- well plates at a density of 1 × 105 cells in 1 ml DMEM-F12. The virus particles (MOI = 50) were added along with 6 *μ*g/ml of Polybrene (Sigma-Aldrich, St. Louis, MO), followed by shaking on a rotator at 5 rpm for 18 hours. The medium was renewed by DMEM-F12. The flow cytometry was applied to calculate the GFP-positive transduced hAD-MSCs at 72 hours after transduction and data were analyzed by BD Accuri c6 software.

### 2.5. Charachterization of Normal/Transduced hAD-MSCs by Differentiation, Flow Cytometry, and CFU-F Assay

In this step, 2 × 10^4^ cells/ml of third passage RIL- 23R-transduced hAD-MSCs and hAD-MSCs were poured in a 6-well plate. The incubation was performed with *α*-MEM containing 10% FBS until reaching confluence. The medium was renewed by osteogenic medium consisting of DMEM having 10 nM of dexamethasone (Sigma-Aldrich, St. Louis, MO, USA), 50 *μ*g/ml of ascorbic acid 2-phosphate (Sigma-Aldrich, St. Louis, MO, USA), and 10 mM of *β*-glycerol phosphate (Sigma-Aldrich, St. Louis, MO, USA). The action was done twice a week for three weeks. Next, the cells were fixed with 10% formalin for 10 min and stained with alizarin red (Sigma-Aldrich, St. Louis, MO, USA) at room temperature for 2 min. In the adipogenesis, the third passage RIL-23R-transduced hAD-MSCs and hAD-MSCs cells were incubated in the presence of differentiation medium containing DMEM with 50 *μ*g/ml of indomethacin (Sigma-Aldrich, St. Louis, MO, USA) and 100 nM of dexamethasone (Sigma-Aldrich, St. Louis, MO, USA). The medium was renewed twice a week for three weeks. The cells staining was performed with 0.5% oil red O (Sigma-Aldrich, St. Louis, MO, USA) in methanol for 2 min at room temperature.

The trypsinization of cells after third passage was carried out by 0.25% trypsin and 0.02% EDTA. The washing was continued twice using PBS, and staining was based on the manufacturer's instruction for running the flow cytometry. The antibody-labeled cells had the antibodies (e-Bioscience) of [1]: PE-conjugated mouse anti-human CD11b, PE-conjugated mouse anti-human CD34, PE-conjugated mouse anti-human CD45, PE-conjugated mouse anti-human CD105, and PE-conjugated mouse anti-human CD73. PE-conjugated mouse IgG1, FITC-conjugated mouse IgG2, and PE-conjugated rat IgG2 were considered to be isotype controls. The incubated cell suspensions were rinsed with PBS to discard any unlabeled antibodies, followed by suspension of labeled cells in PBS. The analysis was done by FACScan flow cytometer (Becton Dickinson, San Diego, CA, USA). The data were analyzed by BD Accuri c6 software.

The self-renewing feature of the cells can be evaluated by Colony-forming unit-fibroblast (CFU-F) assay. 1x10^3^ cells at third passage were seeded in 10 cm dishes. Following cultivation for 2 weeks, the cells were washed with PBS and stained with 0.5% crystal violet (Sigma-Aldrich, St. Louis, MO, USA) for 5 min at room temperature. Stained colonies were counted.

### 2.6. Stemness Markers Expression in Normal/Transduced hAD-MSCs

The expression of OCT-4, sox2, Nanog, and c-Myc was examined in hAD-MSCs and transduced hAD-MSCs by RT-PCR. The GAPDH expression was the endogenous reference gene.

### 2.7. RIL- 23R Expression in Normal/Transduced hAD-MSCs

The RIL- 23R expression was studied in hAD-MSCs and transduced hAD-MSCs. The GAPDH expression was the endogenous reference gene. Qiazol lysis reagent (Alameda, CA, United States) was used to isolate total RNA from the cells. The reactions of standard reverse transcription were done using 5 *μ*g of total RNA by oligo (dT) 18 as a primer according to the manufacturer's instructions for the cDNA synthesis kit (Fermentas). Additional PCR components were 2.5 *μ*l of cDNA, 1 X PCR buffer (AMS), 200 *μ*M of dNTPs, 0.5 *μ*M of each primer pair, and 1 unit per 25 *μ*L of reaction mix Taq DNA polymerase (Fermentas). The above-mentioned pairs of primers detected the expression of RIL- 23R gene.

Whole cell lysate of normal/transduced hAD-MSC was generated from confluent cell layers in 6-well plates using lysis buffer made of 50 mM Tris-Cl, pH 7.4; 150 mM NaCl, 1% NP-40, 1% Na-deoxycholate, 1 mM EDTA, 0.1% SDS (all from Sigma), and Mini Complete, EDTA-free protease inhibitor cocktail (Roche). Proteins were separated by 12% SDS-PAGE, blotted to nitrocellulose membranes (GE Healthcare, Munich, Germany), blocked with TBS containing 5% nonfat dry milk, and analyzed for RIL-23R with the anti-RIL-23R antibody (R&D systems) followed by anti-goat HRP conjugate (Santa Cruz, Santa Cruz, CA, USA).

### 2.8. RIL- 23R Function Assay by Co-Culturing

The IL- 23R secretion and function were investigated by bioassays of naïve T cells of C57BL/ 6 mouse co-cultured with normal / transduced hAD-MSCs and HT1080 cells. The mice were killed in accordance with the laboratory animal protocol to dissect the spleen for digesting with 100 u /ml of dispase/collagenase. The spleen cells were gathered via centrifugation at 1200 rpm and the RBCs lysis via an RBC lysis buffer. The cells were then suspended in RPMI culture medium supplemented with 10% fetal calf serum at a concentration of 1×10^6^ cells/ml. To generate short-term cultures of activated T cells, splenocytes were activated by the addition of the lectin phytohaemagglutinin (PHA) at a concentration of 1 mg/ml in RPMI supplemented with 10% fetal calf serum for 4 days [32]. The cells were then washed and maintained in interleukin-2 (IL-2) supplemented media at a concentration of 2 ng/ml for at least 7 days before use.

2 × 10^4^ cells/well were seeded in 96-well plates. The next day, cells were washed and preincubated with a mitomycin C (0.4 mg/ml for 5 minutes) [33]. Following treatment, the cells were washed immediately at least three times and incubated in RPMI (Sigma) growth medium. To decide whether transduced cells induced Th2 cells proliferation in results from the upregulated RIL-23R expression, IL-2 dependent PHA activated T cells were added to the culture medium with an effector: target cell ratio of 20:1 [34]. T cell-adherent cell contact was inhibited in some experiments by seeding T cells into cell culture inserts (Becton Dickinson). These inserts incorporate membranes that are transparent and contain 0.4 mm pores which allow free passage of soluble factors but are too small to let cell migration [32]. Supernatants were harvested after 24 or 48 h and concentration of IL-10 and IL-17 determined by RT-PCR [35]. Total RNA extraction was carried out from the T cells cocultured with normal/transduced cells. The expression of glyceraldehyde-3-phosphate dehydrogenase (GAPDH) was considered as an endogenous reference gene.

### 2.9. Ethical Considerations

The present study was conducted with the recommendations in the guide for the Care and Use of Laboratory Animals and was approved by the ethics committee of The Golestan University of Medical Sciences. The human adipose tissue was collected after obtaining informed consent considering the Declarations of the Golestan University of Medical Science, Gorgan, Iran

### 2.10. Statistical Analysis

All statistical analyses were carried out by Graph Pad Prism (version 6) using one-way ANOVA and independent t-test to check the differences between groups at the statistical significant level of P < 0.05.

## 3. Results

### 3.1. hAD-MSCs Were Isolated and Differentiated into Adipocytes and Osteoblasts

**Figure 1:**
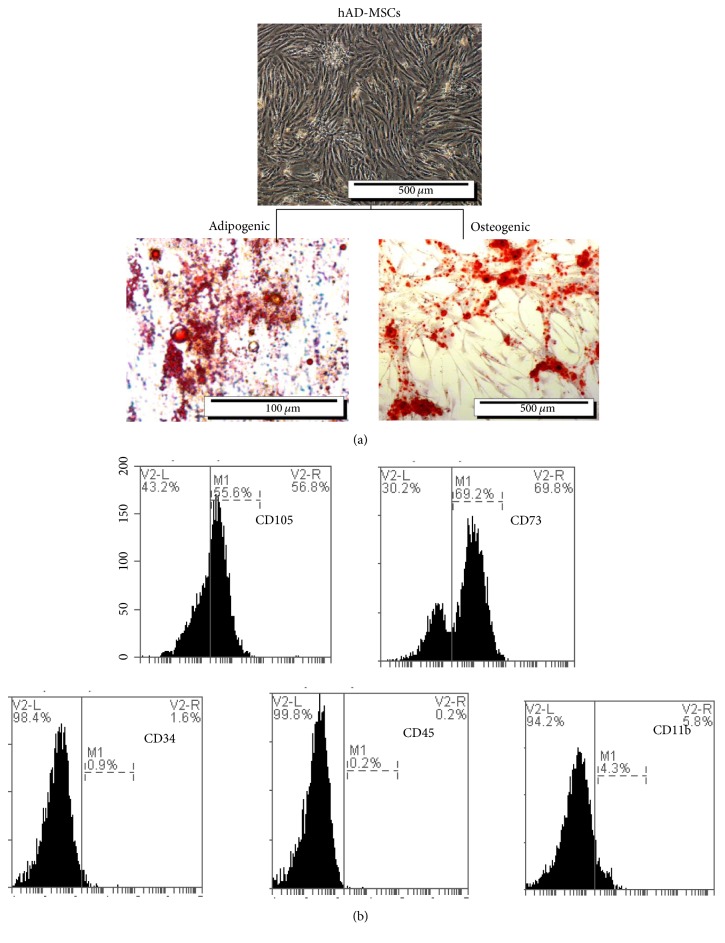
The isolated mesenchymal stem cells (MSCs) characterization by flow cytometry and differentiation. (a) The second passage of isolated hAD- MSCs. Adipogenic: staining of isolated hAD- MSCs after 2nd passage by Oil Red O reagent. Osteogenic: staining of isolated hAD-MSCs by Alizarin Red S reagent. (b) Expression of surface antigens in isolated hAD-MSCs as determined using flow cytometry.

After the cells were passaged, the adipocytes were separated from lipoaspirate tissue using mechanical and enzymatic digestion. The cells displayed a strong proliferative ability (Figure 1). The confirmation of the cell multilineage capacity was done following their differentiation into adipocyte and osteoblast cells after three weeks. Visualization of many lipid vacuoles after oil red staining revealed the adipocyte features of hAD-MSCs (Figure 1(a)). We identified the presence of cell surface markers CD73, CD105 and the absence of hematopoietic (CD45, CD11b) and endothelial (CD34) antigens in isolated adipose MSC cells. Our data show that this type of cell has similar expression profiles for the selected markers (Figure 1(b)).

### 3.2. RIL- 23R Was Designed and Successfully Cloned into a Lentiviral Vector

**Figure 2:**
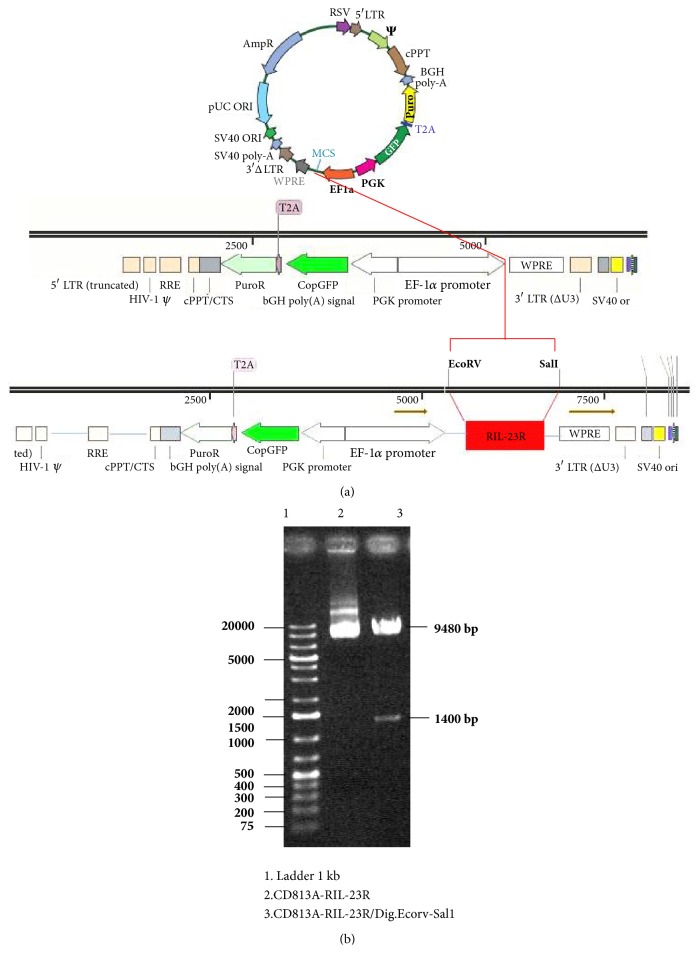
Recombinant IL-23R gene construct. (a) Recombinant IL-23R cDNA inserted into the pCDH-813A-1 lentiviral vector. (b) The presence of a 1400 bp segment related to RIL- 23R and a segment of a 9480 bp related to pCDH-813A-1 confirmed that the cloning established. A genetic map confirmed these data.

The designed recombinant open reading frame of the IL- 23R gene (RIL- 23R) was amplified using PCR and then subcloned into a lentiviral vector (Figure 2(a)). The insertion of RIL-23R cDNA into the pCDH813A-1 vector was confirmed by digestion of the shuttle vector by EcorV and Sal1. The 1400 bp fragment corresponding to RIL-23R was successfully separated from the pCDH813A- 1 vector (Figure 2(b)). Moreover, the colony-PCR was used to detect RIL-23R cDNA in this vector. The cDNA then was sequenced, and no mutations were found in the cDNA sequence.

### 3.3. The Recombinant Viral Particle Was Produced by HEK-293T

**Figure 3:**
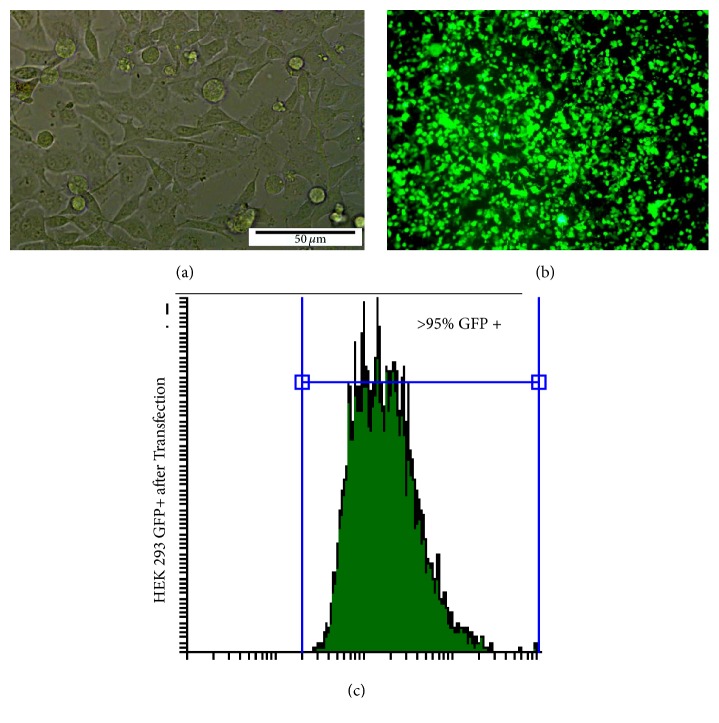
Transfection of HEK-293T for recombinant viral particles production. (a) The nontransfected culture of HEK-293T. (b) Transfected HEK- 293T at 24 hours after transfection by pCDH- EF1*α*- RIL- 23R- PGK-copGFP. (c) Flow cytometry analysis shows High expression of GFP in HEK- 293T and the high rate of transfection.

HEK- 293T cells with 70% confluency (Figure 3(a)) were cotransfected with this construct and successfully produced RIL- 23R- lentiviral particles (Figure 3(b)). The transfection efficiency was over 90% (Figure 3(c)).

### 3.4. hAD-MSCs Were Transduced with Recombinant Lentiviral Particles

**Figure 4:**
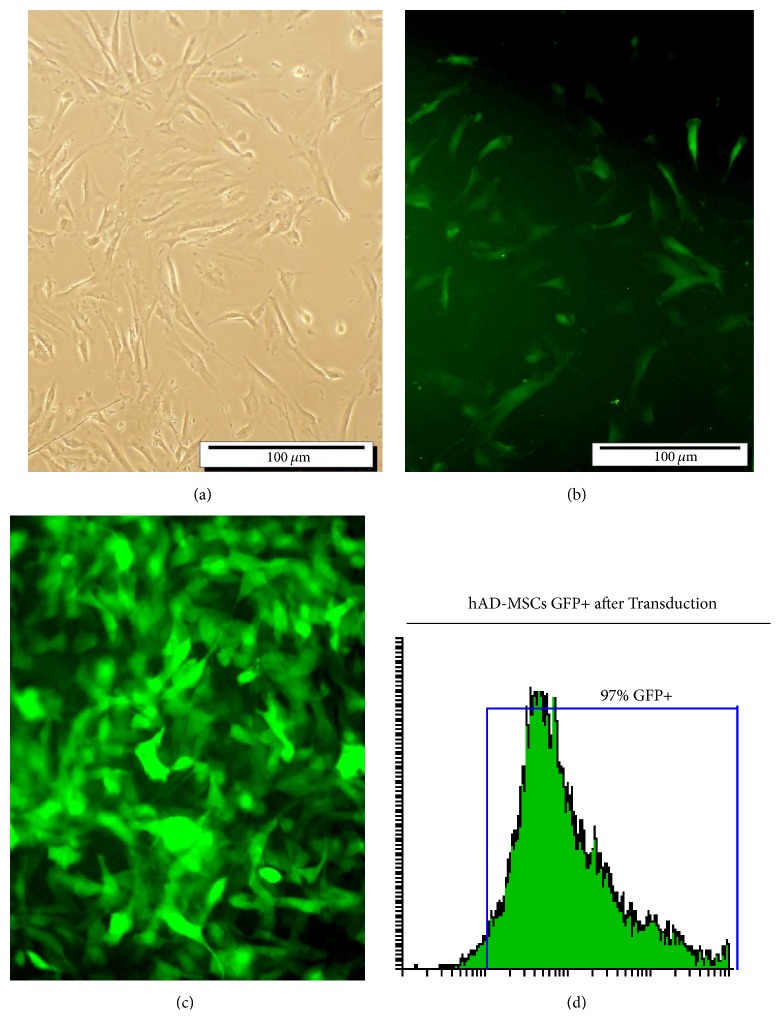
Adipocyte-derived mesenchymal stem cells (hAD-MSCs) transduction with lentiviral particles. (a) hAD-MSCs before transduction. (b) hAD-MSCs transduced with pCDH-RIL-23R-PGK-copGFP lentiviral vector. (c) Transduced hAD-MSCs after puromycin selection; abundant green cells and GFP expression exhibit high transduction level (d) FACS analysis of the transduction rate after puromycin selection indicated that more than 90% of the cells were GFP positive.

AD-MSCs were about 50% confluent when transduced with lentiviral particles (Figure 4(a)). GFP-positive transduced hAD-MSCs measured at 72 h after transduction (Figure 4(b)) and were selected for with 2.5 *μ*g of puromycin (Figure 4(c)). Flow cytometry was used to get about 95% pure transduced cells 14 days post-transduction (Figure 4(d)). The transduced hAD-MSCs at MOI = 50 was conducted after doing virus concentration and titration.

### 3.5. Normal/Transduced hAD-MSCs Were Characterized by Differentiation, Flow Cytometry, and CFU-F Assay

**Figure 5:**
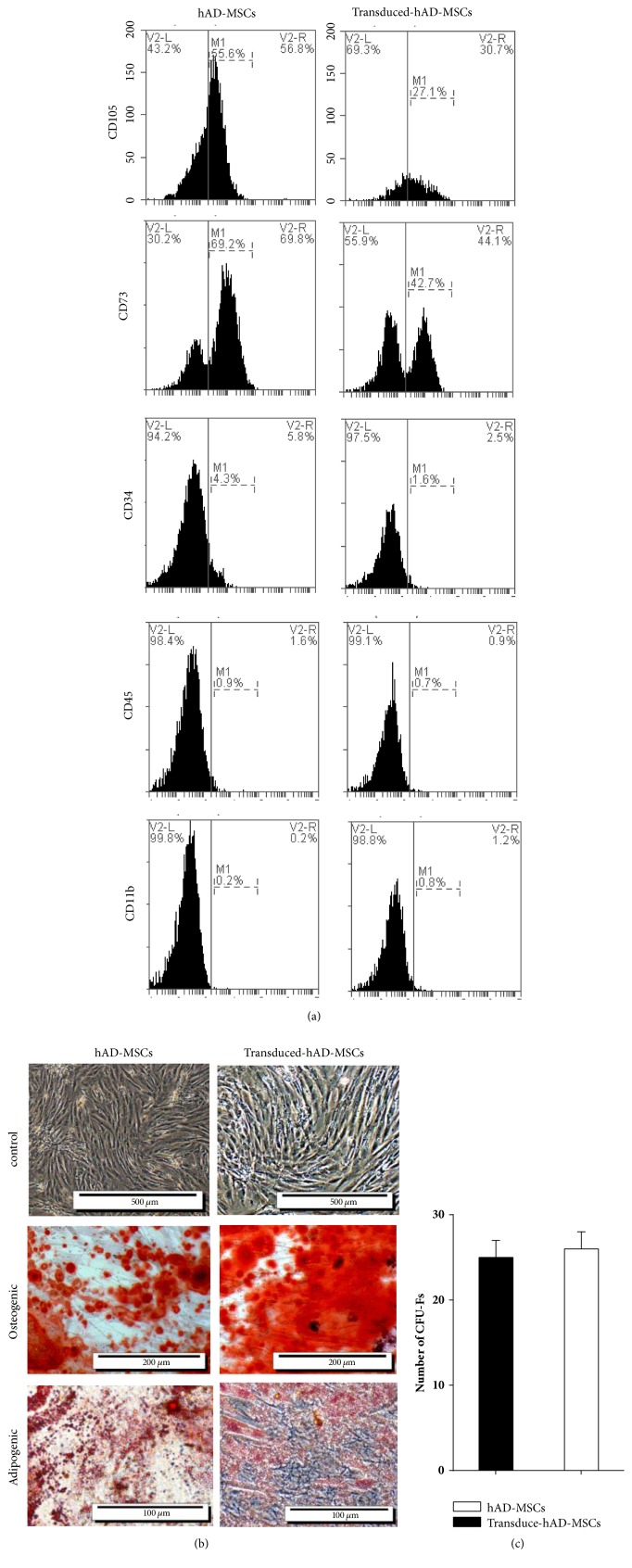
The mesenchymal stem cells (MSCs) characterization by flow cytometry, differentiation, and CFU-F assay. (a) Expression of surface antigens in hAD-MSCs, and transduced hAD-MSCs as determined using flow cytometry. (b) Control: the second passage of hAD- MSCs and transduced hAD-MSCs. Osteogenic: staining of hAD-MSCs and transduced hAD-MSCs by Alizarin Red S reagent; calcium deposits stained bright orange-red around hAD-MSCs differentiated into osteocytes on the 21th day. Adipogenic: staining of hAD-MSCs and transduced hAD-MSCs after 2nd passage by Oil Red O reagent; intracellular vesicles of oil accumulated and stained bright red within hAD-MSCs differentiated into adipocytes on the 21th day. (c) Clonogenic capacity was measured by colony forming unit-fibroblast (CFU-F) assay. The results are represented as the means ± SD.

We identified the presence of cell surface markers CD73 and CD105 and the absence of hematopoietic (CD45, CD11b) and endothelial (CD34) antigens in both hAD-MSCs and transduced hAD-MSCs (Figure 5(a)). Our data show that these two types of cells have similar expression profiles for the selected markers. But the stem cell marker CD105 was less abundant in transduced hAD-MSCs (55.6%  ±4.39 SEM, n=5 for hAD-MSCs, and 27.1%  ±2.3% SEM, n=5 for transduced-hAD-MSCs).

Although mesenchymal stromal cells have been defined by the positive expression of CD105 [36], several groups have also observed considerable phenotypic drift within ASCs during in vitro expansion [37–39]. We found that CD105 expression on hMSCs is heterogeneous in agreement with previous studies [36, 40–42]. The differences observed in CD105 expression could be a consequence of culture conditions (passage number, culture time, cell confluence [43, 44], oxygen pressure, TNF-*α* [45], IFN-*γ*[4], and Serum-Free Medium) [46] and MSC source [47]. Also, Absence or low expression of Endoline (CD105) correlated with a subgroup of adipose-derived cells with increased osteogenic gene expression [36, 48], while the selection of CD105 positive (CD105+) MSCs favors chondrogenesis [49]. These results also showed that the MSCs were not derived from endothelial or hematopoietic cells.

The normal/transduced hAD-MSCs displayed a strong proliferative ability. The cell multilineage capacity was done following their differentiation into adipocyte and osteoblast cells (Figure 5(b)). Visualization of massive calcium depositions around differentiated cells after alizarin Red staining confirmed the presence of osteoblasts in hAD-MSCs and transduced hAD-MSCs. Moreover, observation of many lipid vacuoles after oil red staining revealed the adipocyte features of hAD-MSCs and transduced hAD-MSCs. Therefore, lentiviral particles containing RIL-23R keep the mesodermal properties of hAD-MSCs.

The CFU-F assay was performed to check the self-renewing properties of the cells. There were no significant differences in the number of CFU-Fs following seeding cells in 10 cm dishes after 2 weeks (Figure 5(c)). The normal and transduced hAD-MSCs displayed a higher self-renewal feature regardless of growth rate, although the differences were not significant.

### 3.6. Self-Renewing Features of hAD-MSCs Were Analyzed by RT-PCR

**Figure 6:**
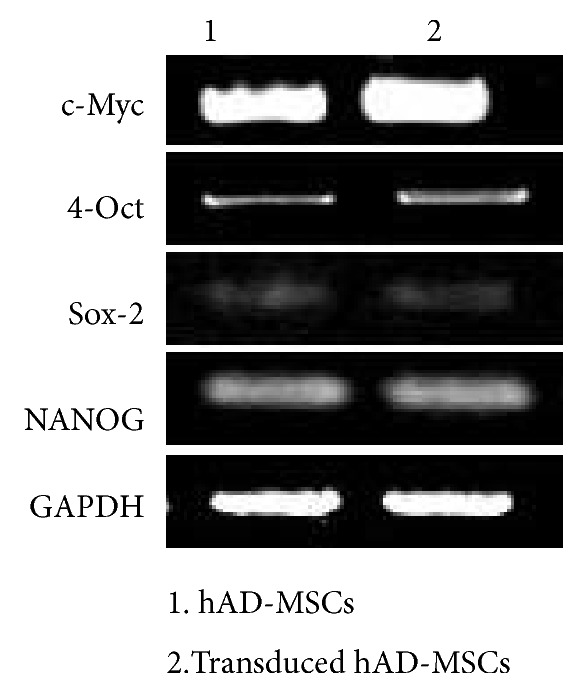
Stemness markers expression in hAD-MSCs and transduced hAD-MSCs.

We examined the expression of molecular markers in the hAD-MSCs and transduced hAD-MSCs (Figure 6). The OCT4, c-Myc, SOX2, and NANOG were detected in the hAD-MSCs and transduced hAD-MSCs. the expression of SOX2 was low in both cell lines. These results suggest that hAD-MSCs and transduced hAD-MSCs have the highest capacity for self-renewal and differentiation potential. Primer sets used for RT-PCR was shown in Table 1.

### 3.7. RIL- 23R Expression Were Analyzed by RT-PCR and Western Blot

**Figure 7:**
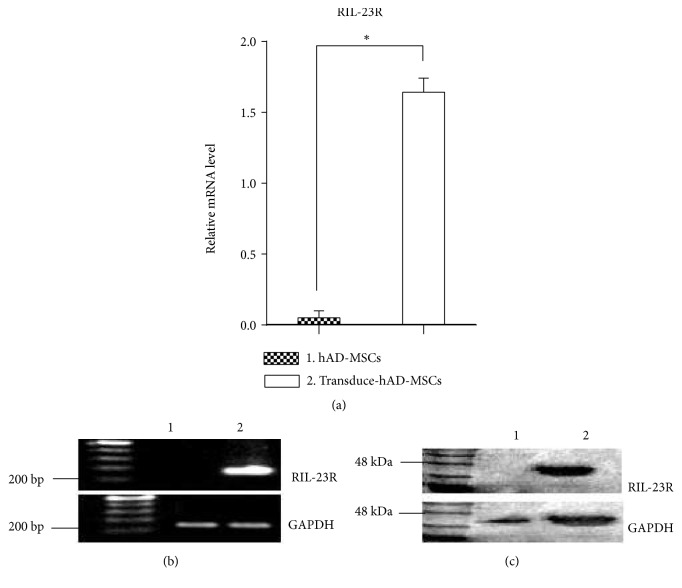
Expression of RIL- 23R in hAD-MSCs and transduced hAD-MSCs. (a-b) qPCR analysis of the RIL-23R in hAD-MSCs and transduced hAD-MSCs. the relative copy numbers of RIL-23R in transduced hAD-MSCs were compared with the copy number in hAD-MSC. The data are shown as means ± SD of 5 independent experiments. (c) Western blot analysis of the RIL-23R in whole cell lysates of hAD-MSCs (20 *μ*g protein was loaded per lane).

For RIL-23R expression, total RNA of nontransduced and transduced hAD-MSCs were isolated, and RT- PCR showed expression of RIL-23R in transduced hAD-MSCs but not in control hAD-MSCs (Figures 7(a) and 7(b)). Subsequently, the expression of RIL-23R was determined on the protein level. By immunoblot analysis, RIL-23R was almost detectable in whole cell lysate of transduced hAD-MSCs (Figure 7(c)).

### 3.8. RIL- 23R Function Was Assayed by Co-Culture with CD4+ T

To prove that the inhibitory effect of hMSCs on murine T cells is specific to the transduced h-ADMSCs, we also used a different adherent human cell line in our experiments, HT-1080 (human fibrosarcoma cell line) [50]. These cells have similar morphological features to hMSCs, without having any known MSC-like properties (the multi-potent ability to differentiate into osteocytes, chondrocytes and adipocytes); also the fibroblasts do not have any inhibitory effect on the T cells [50], therefore, they were used as controls in our experiments. We used these cell lines in the same ratios as the hAD-MSCs and the same experimental settings.

## 4. Discussion

Autoimmune disease occurs when body organs are attacked by autoimmune cells as a result of an unfit immune response directed to autoantigens [51]. The most immunosuppressive drugs for the treatment of autoimmune disease belong to the corticosteroids family [52]. Many medical investigators are seeking new immunotherapeutic strategies with fewer side effects. These strategies have included a gene or recombinant protein [53] therapies for affecting specific immune cells [54] and molecules such as cytokines [55], chemokines [56], and costimulatory molecules [57].

Mesenchymal stem cells (MSCs) are known to have immunomodulatory, self-renewing, and multilineage differentiation properties [58]. These characteristics have led to recognition of the true capacity of MSC-based cell therapy. Due to the efficiency with which MSCs can be transduced with different genes [59], genetic modifications of these cells have been carried out to enhance MSC efficacy in tissue repair/regeneration [9].

The high amount of MSCs found in adipose tissues and the relative ease with which they can be isolated makes MSCs a good source of adult stem cells in regenerative medicine [19]. Some studies [12, 13] have shown that manifestly more stem cells can be obtained from adipose tissue compared to the same volume of bone marrow [60].

In the present research, the hAD-MSCs were successfully isolated from lipoaspirate tissue samples and characterized by flow cytometry. As well, their osteogenic and adipogenic differentiation were analyzed. All transduced and non-transduced cultures were able to differentiate into adipogenic and osteogenic lineages, to express stem cell markers such as OCT- 4, SOX2, NANOG and c-Myc retain the nature of hAD-MSCs. There were no significant variations in proliferation capacity or cell surface marker expression between transduced and non-transduced cultures. There was a decrease in CD105, an MSC-specific marker. The transduced and non-transduced cells showed the same morphologies and differentiation [61].

Endoline (CD105), the TGF-b receptor III, is generally considered an important marker for MSCs [62]. We have reported that the stem cell marker CD105 was less abundant in transduced hAD-MSCs. several reports have shown that its expression varies depending upon MSC source, culture time in vitro, and differentiation state [40, 63].

Anderson et al. [40] demonstrated that both CD105- and CD105+ mASCs had a similar proliferative capacity, colony-forming unitfibroblast (CFU-F) potential, and expression of differentiation-related genes and shared all other MSC markers analyzed. CD105-mASCs had a greater capacity to differentiate into adipocytes and osteocytes also were more efficient at inhibiting T cell proliferation in vitro compared to CD105+ mASCs. When the mASC cultures reached confluency, membrane type 1 matrix metalloproteinase, which is a membrane-tethered MMP, can cleave CD105 from the cell surface [64]. Levi et al. [36] found that expression patterns for CD105 to be closely associated with the osteogenic potential of ASCs. Although they cannot necessarily predict the nature of these differences, with respect to CD105 and its role as a coreceptor for TGF-*β*1, they would expect that CD105 low cells would show reduced TGF-*β*1 signaling.

The natural form of IL-23R is encoded by at least 12 exons. Here, we designed recombinant IL-23R (RIL-23R) that can produce a secreted form of the receptor with an antagonistic function against IL-23 [29]. Some studies have shown that the IL-12R*β*2 chain or IL-23R exhibited tumor suppressor functions [65]. In addition, evidence indicates that soluble receptors play roles as agonists and antagonists during disease and normal homeostasis [66]. Therefore, we propose that generation of this soluble receptor might give a method of immune maintenance in autoimmune diseases.

The MSCs can be used as a gene delivery system because of their ability to be easily transformed and home in on injured tissues and their lack of immunogenic properties. Additionally, they have been transduced with different vectors to optimize transgene expression [9]. In some cases, such as therapy for long-life illnesses, permanent transduction of MSCs may be needed and can be attained by using retroviruses to change MSCs with high efficiency to get long-term expression [67].

We obtained more than 95% lentiviral transduction efficiency of AD-MSCs according to the selection of puromycin as a beneficial agent for ideal therapeutic purposes [68].

The RT-PCR verified expression of RIL- 23R. The present findings and other those from similar studies found no negative effects on multipotency properties after transgene expression by lentiviral transduction [30].

The IL-23 is effective in the function of Th17 cells but unable to induce the differentiation of these cells ***in vitro***. The ability of the soluble isoform of IL-23R to inhibit IL-23 signaling [69] led us to hypothesize that our RIL-23R, acting as an IL-23 inhibitor, may bind the p19 chain of IL-23 and inhibit the functional maturation of Th17 cells, resulting in T cell production leading to large amount secretion of IL-10 in a shift to the Th2 cell type [70]. Then, we expected overexpression of IL-10 and suppression of IL-17. The RT-PCR is capable of exhibiting the expression level of the target genes; however, bioassays exactly confirmed the potency of gene expression. The stem cells have some features of cancer cells including long lifespan, relative apoptosis resistance, and ability to replicate for extended periods of time [71]. In addition, similar growth regulators and control mechanisms are involved in both cancer and stem cell maintenance [72]. STS cell lines harbor differentiation capacity similar to MSCs [73]. Recent evidence strongly suggests that sarcomas originate from mesenchymal stem cells [74–77], which represent a plastic-adherent spectrum of different bone marrow-derived cells until recently. Identically treated cultures of the highly metastatic fibrosarcoma cell line HT1080 were included as positive controls. We isolated mouse CD4+T-cells from splenocytes and cocultured them for three days in the presence of the transduced hAD-MSCs and HT1080 cells and hAD-MSCs and HT1080 cells as control cells. The ability of our recombinant RIL-23R was also evaluated to express IL-17 and IL-10 by RT-PCR. Our RIL-23R induced the Th2 cell proliferation and improved their function by IL-10 overexpression [74].

## 5. Conclusions

It was the first report of RIL-23R transduction into hAD-MSCs, which may be an effective approach to use such cells as a good vehicle for cell-based gene therapy in autoimmune disorders.

## Figures and Tables

**Table 1:** Primer sets used for RT-PCR.

Gene	Primer sequence (5′ → 3′)	Product size (bp)
c-Myc	Forward: TCGGATTCTCTGCTCTCCTC	413
	Reverse: CGCCTCTTGACATTCTCCTC	

4-Oct	Forward: GACAACAATGAGAACCTTCAGGAGA	218
	Reverse: TTCTGGCGCCGGTTACAGAACCA	

SOX2	Forward: AACCAAGACGCTCATGAAGAAG	341
	Reverse: GCGAGTAGGACATGCTGTAGGT	

NANOG	Forward: ATAGCAATGGTGTGACGCAG	219
	Reverse: GATTGTTCCAGGATTGGGTG	

GAPDH	Forward: GTGGTCTCCTCTGACTTCAACA	210
	Reverse: CTCTTCCTCTTGTGCTCTTGCT	

## Data Availability

The data used to support the findings of this study are included within the article.
